# INNOVATION AND SUSTAINABILITY IN PATIENT CARE IN THE ERA OF HEALTH RESOURCE MOBILIZATION

**DOI:** 10.1590/S0004-2803.24612025-E02

**Published:** 2026-03-23

**Authors:** Everson Luiz de Almeida ARTIFON, Alberto TERRIVEL, Marcio Roberto FACANALI

**Affiliations:** 1 Hospital das Clínicas, Faculdade de Medicina da Universidade de São Paulo, Departamentode Cirurgia, São Paulo, SP, Brasil.; 2 Instituto Vita, Departamento de Medicina do Esporte, São Paulo, SP, Brasil.; 3 Universidade de São Paulo, Departamento de Gastroenterologia, São Paulo, SP, Brasil.

## Contemporary challenge: sustainability in healthcare

Modern medicine faces a paradox. While technology, science, and digital integration advance rapidly, the field simultaneously faces growing financial constraints and unequal access to care. In Brazil, chronic underfunding of the Unified Health System (SUS) and dependence on short-term or emergency funding threaten institutional sustainability and the continuity of innovation.

This duality calls for a strategic and ethical perspective: to innovate with purpose, ensuring that every investment generates clinical, scientific, and social value. Sustainability, therefore, is not only financial but also organizational, human, and systemic, reflecting the ability of institutions to maintain innovative practices over time^1^.

## Innovation with purpose: the new axis of sustainability

“Innovation” is no longer synonymous with adopting new technologies. It now represents the creation of sustainable value, improving patient care, reducing waste, and strengthening healthcare outcomes. The challenge lies in translating technological progress into measurable impact while considering cost, safety, and accessibility.

Recent conceptual models demonstrate that the sustainability of healthcare innovations depends on three fundamental elements: institutional alignment, continuous adaptation, and public policy support^2^. The success of an innovation is not defined by its implementation alone, but by its capacity to produce consistent and reproducible results over time.

In gastroenterology, this principle applies from the responsible use of artificial intelligence in colonoscopy to the development of structured robotic and simulation-based training programs. Each innovation must be grounded in evidence, cost-effectiveness, and institutional commitment.

## Resource mobilization and institutional accountability

Today, sustainability is directly linked to the ability to secure and efficiently manage financial resources. University hospitals and research centers in Brazil have shown that it is possible to integrate science, care delivery and financial responsibility, transforming funding into instruments of social innovation and public system strengthening.

However, the literature emphasizes that financial sustainability is fragile when detached from long-term planning and transparency. In Brazil, chronic underfunding and budgetary rigidity remain structural barriers to achieving the targets of the 2030 Agenda^3^. For this reason, funding initiatives must be accompanied by sound governance, impact assessment, and collaboration between academia, the public sector, and private partners^4^.

## From isolated investment to a sustainable ecosystem

Sustainability in healthcare cannot be achieved through isolated actions, but rather through the creation of integrated ecosystems of innovation, education, and research.

Thematic innovation centers and simulation units in surgery and endoscopy exemplify how well-directed investments can yield multiple returns, from improved clinical quality and professional training to the generation of new scientific knowledge.

Every investment must ultimately translate into better clinical outcomes and operational efficiency. Building collaborative networks that share resources, data, and infrastructure represents the most reliable path to long-term sustainability and social relevance.

## The physician as an agent of sustainability

The physician of the twenty-first century is not merely a technical executor but an agent of institutional transformation. He or she plays an active role in defining priorities, mobilizing resources, and evaluating outcomes. This cultural transition, from technology consumer to co-creator of innovation, is essential to make the healthcare system more efficient, autonomous, and sustainable.

Training professionals with this interdisciplinary and ethical mindset is an urgent task for universities and medical societies in Brazil.

## CONCLUSION

Innovation and sustainability are not opposing goals but complementary dimensions of the same mission: to improve patient care and preserve the healthcare system for future generations.

Every project must answer a fundamental question: “Does this initiative improve patient care, reduce inequities, and strengthen the public healthcare system?”

When the answer is yes, we are facing a truly transformative innovation.

Sustainability, in this context, becomes not only a financial requirement but an ethical and societal imperative, the cornerstone of a medical practice that unites science, efficiency, and humanity.

## Data Availability

Not applicable - The study did not use research data
